# Fungal Peritonitis Associated With Peritoneal Dialysis Due to Non-Albicans Candida: A Case Series

**DOI:** 10.7759/cureus.32658

**Published:** 2022-12-18

**Authors:** Daniel Juarez Villa, Karla Berenice Cano Escobar, Sebastian Toledo Ramirez, Ivan Zepeda Quiroz

**Affiliations:** 1 Nephrology, Instituto Nacional de Cardiología Ignacio Chávez, Mexico, MEX; 2 Nephrology, Instituto Nacional de Cardiología Ignacio Chavez, Mexico, MEX

**Keywords:** mortality, chronic kidney disease, fungal peritonitis, dialysis peritonitis, case report

## Abstract

Fungal peritonitis secondary to non-*albicans Candida* is reported less frequently. There are uncertainties regarding the treatment of non-*albicans Candida* infection (i.e., preferred route or initial drug). The objective of this study is to determine the clinical characteristics and treatment used in cases of peritoneal dialysis associated fungal peritonitis secondary to non-*albicans Candida*. We report four cases with different clinical characteristics and different routes of administration of the antifungal drug, with no deaths. In all four patients, there were risk factors similar to those reported worldwide, without presenting the route of administration of the antifungal drug as a risk factor, suggesting that the mainstay of treatment is early initiation of the antifungal drug and early removal of the catheter.

## Introduction

Fungal peritonitis occurs in around 1-12% of patients on peritoneal dialysis (PD). It has a poor prognosis as it is usually associated with catheter obstruction, abscess formation, and sclerosing peritonitis. The mortality rate is around 5-53%, and PD catheter malfunction is around 40-55%, resulting in conversion to hemodialysis. Candida species has the highest incidence of PD-associated fungal peritonitis, responsible for approximately 60-90% of cases. On the other hand, non-*albicans Candida *cases are reported less frequently. There are uncertainties regarding treatment of non-*albicans Candida* infection (i.e., preferred route or initial drug), but based on case reports and small case series, fluconazole and amphotericin B are the recommended drugs [1-5].

The objective of this study is to determine the clinical characteristics and treatment used in cases of PD-associated fungal peritonitis secondary to non-*albicans Candida*, as they are less frequently reported and with more controversies in the management. Data were collected retrospectively by reviewing the records of all PD patients with confirmed fungal peritonitis secondary to non-*albicans Candida*, using peritoneal fluid culture from January 2015 to August 2021. Four cases were found during the period.

## Case presentation

Case 1

A 53-year-old female with a body mass index (BMI) of 27.32 kg/m^2^, diagnosed with chronic kidney disease (CKD) stage 5 secondary to hypertensive nephrosclerosis and renal lithiasis for 19 years, was on continuous ambulatory peritoneal dialysis (CAPD) for three years and four exchanges with glucose solution without final infusion per day without residual kidney function. The patient presented with cloudy dialysate but without fever. She had a history of just one peritonitis, six months prior to her latest admission, but without isolation of the microorganism and any other risk factor for peritonitis. Her previous peritonitis was treated with intraperitoneal vancomycin, which resulted in improvement of symptoms. For her most current peritonitis, isolation of *Candida parapsilosis* was noted in peritoneal fluid culture. PD catheter was removed, and she was started on amphotericin 50 mg intravenously daily for 15 days, and after hospital discharge, voriconazole was started at 400 mg daily for a month. She was shifted to hemodialysis. She was eventually discharged but was lost to follow-up.

Case 2

A 30-year-old, male with a BMI of 21.88 kg/m^2^ diagnosed with CKD stage 5 secondary to urinary malformation for 10 years was on automated peritoneal dialysis (APD) with infusion of icodextrin (high transporter). He had a history of kidney transplantation (living-related donor), which lasted for six years, but now with in-graft loss. He had a history of peritonitis six months prior to his condition, without isolation of microorganism on peritoneal fluid culture. Vancomycin and intraperitoneal amikacin were administered for 14 days as the local protocol treatment. For his most recent peritonitis, he presented with generalized abdominal pain with cloudy dialysate without fever. *Candida tropicalis* was isolated on the culture. PD catheter was removed. He was given intraperitoneal fluconazole 200 mg per day for 10 days. During his hospital stay, he developed mechanical ileus that required nasogastric tube and surveillance. He was discharged home stable, and was shifted to hemodialysis.

 Case 3

A 28-year-old female with a BMI of 32 kg/m^2^ presented with a history of CKD secondary to neurogenic bladder for five years; initially, she was on hemodialysis for two years but was eventually shifted to APD with prescription of 10 liters and 10 hours with infusion of 2 liters of 2.5% glucose solution. Two weeks prior to her admission, she was treated for a possible bacterial peritonitis due to abdominal pain and cloudy dialysate but without isolation of organism. She was given imipenem for two weeks in another hospital. The abdominal pain without fever persisted (three weeks), and she was eventually admitted. On repeat peritoneal fluid culture, *Candida stellatoidea* was isolated. PD catheter was removed and fluconazole 200 mg per day was administered intravenously. She was eventually shifted to hemodialysis. After three years, she underwent renal transplantation from a deceased donor.

Case 4

A 60-year-old female with a BMI of 24 kg/m^2^ was diagnosed with CKD secondary to diabetic kidney disease. She started PD because she presented with diuresis of 1 liter per day. One month after the start of PD, she presented with generalized abdominal pain and cloudy dialysis fluid without ever-presenting fever. A cytological examination was performed with evidence of peritonitis, and treatment was given initially with vancomycin and intraperitoneal amikacin. Then, with positive culture for *Pseudomonas aeruginosa*, the treatment was changed to intraperitoneal ceftazidime and oral ciprofloxacin. After five days with the persistence of cytology with 313 cells with 100% of polymorphonuclear, a new culture was requested, which revealed *Candida parapsilopsis* and *Tichosporon*, for which oral fluconazole and oral ciprofloxacin were given, removing the PD catheter and migrating to hemodialysis.

## Discussion

The four cases we reported present risk factors similar to those reported worldwide, such as previous peritonitis and antibiotic use. Also, as risk factor, our patients had albumin less than 3.5 g/dL, which has been documented as an independent risk factor for fungal peritonitis [6]. None of them died, which is important to mention, since mortality worldwide is high. This was derived from early catheter removal and introduction of antifungal drugs after evidence of the microorganism. The mortality in the worldwide is high if the catheter is not removed early (50-91%) [7]. The clinical and laboratory characteristics of the four cases are shown in Table 1.

**Table 1:** Clinical and biochemical characteristics of patients with fungal peritonitis. ++Indicates moderate yeasts by high power field BMI, body mass index; CAPD; continuous ambulatory peritoneal dialysis; APD, automated peritoneal dialysis; HD, hemodialysis

Variable	Case 1	Case 2	Case 3	Case 4
Age (years)	53	30	28	60
BMI (kg/m^2^)	27	21.8	32	24
Gender	Female	Male	Female	Female
History of diabetes or immunosuppression use	None	None	None	None
Previous modality	CAPD 3 years	APD 6 years	APD 3 years	None
Fungal prophylaxis	None	None	None	None
Previous antibiotics	Intraperitoneal vancomycin	Intraperitoneal vancomycin + amikacin	Systemic imipenem	Intraperitoneal vancomycin + amikacin. Later on intraperitoneal ceftazidime plus oral ciprofloxacin
Number of previous peritonitis	1	2	0	0
Isolation	Candida parapsilosis	Candida tropicalis	Candida Stellatoidea	*Candida parapsilosis *and* Trichosporon*
Treatment	Catheter removal plus migration to HD + amphotericin 50 mg daily for 14 days + voriconazole 400 mg daily for one month	Catheter removal plus migration to HD + fluconazole 400 mg single dose intraperitoneal + fluconazole 200 mg intravenous daily for 14 days + fluconazole 200 mg oral for 10 days	Catheter removal plus migration to HD and intravenous fluconazole 200 mg for 14 days	Catheter removal plus migration to HD and oral fluconazole 200 mg for 30 days
Definitive renal replacement therapy	Hemodialysis	Hemodialysis	Hemodialysis	Hemodialysis
Hemoglobin (g/dL)	5.9	9.6	11.1	9.7
Total leucocytes (x10^3^/μL)	14.23	8.45	8.09	9.76
Absolute neutrophils ( x10_3_/μL)	12.35	6.58	6.74	8.61
Platelets (x10^3^/μL)	421	618	289	344
C-reactive protein (mg/L)	164	>300	231	81.5
Glucose (mg/dL)	116	97	112	254
Albumin (g/dL)	3	3.15	3.2	3.03
Leucocyte count in peritoneal fluid cytology (100/µL)	500	93	4700	1265
Polymorphonuclear (%)	99	100	100	100
Gram staining of peritoneal fluid	No Microorganism	Yeast without pseudomyceles ++	No microorganism	No microorganism

It is important to mention that all four patients presented with non-specific symptoms such as abdominal pain, without fever. In the laboratory data, there was no evidence of any significance, only one patient had systemic leukocytosis and only one had yeasts in the Gram stain of peritoneal fluid. Three patients received fluconazole (one given as intraperitoneal) and the other one received amphotericin (11 intraperitoneal and systemic doses). The treatment duration was at least two weeks according to the most recent guidelines [8]. No patient had any extraperitoneal manifestations of fungal infection. This may suggest that the route of administration is not the most important factor in the treatment of fungal peritonitis.

After treatment and cavitary rest, PD was attempted again, without success. In multiple studies, the chance of returning to PD was 40% [9,10].

In our four cases, all of them were converted to hemodialysis, which is one of the main complications of fungal peritonitis. None of our patients died because of the timely removal of catheter and administration of antifungal treatment. These factors could help decrease mortality.

## Conclusions

Fungal peritonitis is an infrequent entity. It has occurred only on four occasions in our center. Although in the literature, the mortality is around 50%, in our cases, none of them died. However, all the patients had to be shifted to hemodialysis. PD catheters were removed in all the four cases. Systemic antifungal treatment was used, and in two cases, intraperitoneal treatment was also added using drugs recommended in the literature.

The mainstay of treatment is prompt catheter removal and early initiation of antifungals. Further studies are needed to determine whether the route of antifungal administration is a protective factor in reducing mortality.
